# Randomised Controlled Trial on the Additive Effect Between Calcium Hydroxide and Sodium Hypochlorite in an Inter‐Visit Root Canal Dressing

**DOI:** 10.1111/iej.70078

**Published:** 2025-12-01

**Authors:** Nidambur V. Ballal, Namith Rai, Padmaja A. Shenoy, Vibha Acharya, Vinutha R. Bhat, Matthias Zehnder

**Affiliations:** ^1^ Department of Conservative Dentistry and Endodontics, Manipal College of Dental Sciences, Manipal Manipal Academy of Higher Education Manipal Karnataka India; ^2^ Department of Microbiology, Kasturba Medical College, Manipal Manipal Academy of Higher Education Manipal Karnataka India; ^3^ Department of Forensic Medicine and Toxicology, Kasturba Medical College, Manipal Manipal Academy of Higher Education Manipal Karnataka India; ^4^ Department of Biochemistry, Kasturba Medical College Manipal, Manipal Academy of Higher Education Manipal Karnataka India; ^5^ University of Zurich Clinic of Conservative and Preventive Dentistry Zurich Switzerland

**Keywords:** apical periodontitis, calcium hydroxide, disinfection, root canal, sodium hypochlorite

## Abstract

**Aim:**

To assess whether there was an additive antimicrobial effect between calcium hydroxide (Ca(OH)_2_) and sodium hypochlorite (NaOCl) in an inter‐visit root canal dressing.

**Methodology:**

Patients presenting with a single‐rooted tooth with pulpal necrosis and asymptomatic apical periodontitis were included in this randomised controlled single‐center clinical superiority trial with two parallel arms. Teeth were instrumented using a rotary file system and irrigated with 3% NaOCl. Subsequently, the root canals were dressed with a Ca(OH)_2_ slurry that was prepared from pure Ca(OH)_2_ powder mixed with either 3% NaOCl (test group) or physiological saline solution (control group). Microbial samples were obtained after accessing the root canal, after chemo‐mechanical preparation, and after the root canal dressing with the test or control slurries. A periapical fluid sample was collected after the root canal dressing removal. Samples were transferred to the microbiology lab immediately and assessed for anaerobic growth. The levels of MMP‐9 were measured using a specific enzyme‐linked immunosorbent assay. Negative to positive growth ratios were compared between groups using Fisher's exact test. Colony‐forming units (CFUs) between and within groups and MMP‐9/total protein were compared using non‐parametric tests, *p* < 0.05.

**Results:**

From the 110 patients recruited for this study, 48 were available for all three sampling procedures in the test (NaOCl) and 50 in the control (saline) group. All canals showed anaerobic growth initially. Chemo‐mechanical instrumentation significantly reduced CFU counts (*p* < 0.001), yet all canals remained growth‐positive. After dressing with Ca(OH)_2_/NaOCl, 19 of the 48 (40%) root canals were free of growth, compared to 3 of 50 (6%) in the Ca(OH)_2_/saline group (*p* < 0.001). MMP‐9/TP levels in the periapical fluid were similar when root canals were dressed with Ca(OH)_2_ slurries mixed with NaOCl or saline (*p* > 0.05).

**Conclusions:**

Placing a combined NaOCl/Ca(OH)_2_ dressing significantly reduced the microbial load in the root canals of teeth with primary apical periodontitis compared to a conventional slurry prepared with saline solution, without causing any apparent inflammatory response in the periapical tissues.

**Trial Registeration:** CTRI/2020/10/028484 (Clinical Trials Registry India)

## 1 Introduction

The aim of chemo‐mechanical root canal debridement during a pulpectomy is to reduce pulp remnants and biofilm as thoroughly as possible. To dissolve these organic entities, alkaline biolytic agents can be applied: sodium hypochlorite (NaOCl) in aqueous solution as an irrigant and/or a calcium hydroxide (Ca(OH)_2_) preparation as an interim dressing (Hasselgren et al. 1988; Tawakoli et al. 2017). In theory and in an ex vivo environment, each of these agents can replace the other (Türkün and Cengiz 1997; Zehnder et al. 2003). NaOCl is used as an irrigating solution (Dutner et al. 2012). It is fast‐acting and used up within 1 h if passively placed in the root canal system (Ragnarsson et al. 2015). NaOCl solutions are therefore especially useful in single‐visit endodontic treatments. Ca(OH)_2_, on the other hand, is applied either as a powder in aqueous suspension or as a premixed formula of the powder in polyethylene glycol (Alnæs et al. 2024). It exerts a slow‐onset but long‐lasting proteolytic/antimicrobial effect (Sjögren et al. 1991; Zehnder et al. 2003).

Clinical studies have shown that a Ca(OH)_2_ dressing may not be necessary after thorough cleaning and shaping of the root canal system with copious NaOCl irrigation (Weiger et al. 2000; Sathorn et al. 2007). Under these conditions, Ca(OH)_2_ appears to merely prevent the re‐growth of microorganisms (Sathorn et al. 2007). On the other hand, if a chemically inert irrigant is used during root canal preparation, a Ca(OH)_2_ dressing applied as an inter‐visit dressing for 1 week can have a remarkable antimicrobial effect (Ørstavik et al. 1991). Moreover, research in infected single‐rooted human teeth has shown that each step taken towards reducing the intracanal bioburden, including the application of an inter‐visit Ca(OH)_2_ dressing, can have an individual or at least a carry‐over effect (Carvalho et al. 2020). In other words, the more time and effort spent on chemical debridement of the canal system, the better. In a single‐visit approach, this can involve activation of the sodium hypochlorite solution (Meire and de Moor 2024), or the use of specific tools to clean the canal walls more efficiently (Emara et al. 2021; Rodrigues et al. 2015). These tools may reduce the chair‐time that is necessary to reach the cleaning and shaping goal defined above. However, treating root canals in a single visit is not always possible or reasonable (Mergoni et al. 2022).

An alternative approach for more effective chemical root canal treatment in two visits could be to combine chemicals, that is, the short‐lived but intense NaOCl effect with the slow‐onset but sustained Ca(OH)_2_ counterpart. NaOCl solutions are naturally alkaline, and their proteolytic power increases with the addition of extra alkali salts such as NaOH (Jungbluth et al. 2011) or Ca(OH)_2_ (Zehnder et al. 2003). Ca(OH)_2_ maintains its properties when mixed with NaOCl and vice versa (Haenni et al. 2003). Mixing calcium hydroxide powder with a NaOCl solution could thus save chair time in a two‐visit clinical approach if there were an additive clinical effect between these two components. This should be expected based on data from extracted and artificially infected bovine teeth (Zehnder et al. 2003). Similar to the “walking bleach” technique in intradental tooth whitening (Spasser 1961), the passive placement of the NaOCl solution could be executed without using the dental chair (Ragnarsson et al. 2015). However, a clinical study revealed no synergy when Ca(OH)_2_ powder was mixed with a 2% chlorhexidine solution (Zerella et al. 2005). Chlorhexidine and Ca(OH)_2_ are chemically non‐compatible, which may explain the result (Haenni et al. 2003).

The aim of this randomised trial was to assess whether there was an additive effect between NaOCl and Ca(OH)_2_ in clinics. We tested the short‐term impact of admixing a 3% NaOCl solution to Ca(OH)_2_ powder in single‐rooted teeth in adult patients with primary non‐symptomatic apical periodontitis treated in two visits. The control treatment was to place a conventional Ca(OH)_2_ dressing prepared with sterile physiological saline solution for the interim. The primary outcome was the proportion of negative anaerobic microbial cultures after 1 week of placing these inter‐visit dressings. The null hypothesis tested was that preparing a Ca(OH)_2_ slurry with a 3% NaOCl solution did not result in more negative cultures compared to a standard slurry prepared with sterile physiological saline solution. The evolution of viable anaerobic counts over the course of the treatment was also monitored. To study possible inflammatory effects by the NaOCl in the periapical tissues as a secondary outcome, periapical fluid samples were taken at the second visit and assessed for a molecular marker (MMP‐9) associated with neutrophil activity (Wahlgren et al. 2002; Ballal et al. 2019).

## 2 Methods

### 2.1 Study Design

This was a randomised controlled single‐center clinical superiority trial with two parallel experimental arms. The trial was approved by the institutional ethics committee (118/2020) and registered at Clinical Trials Registry India (CTRI/2020/10/028484 [Registered on: 20/10/2020]). All patients were informed regarding the benefits, risks, and alternative treatment choices before enrollment in the trial. They were furthermore told that not participating in this study had no consequences regarding their treatment. Informed consent was obtained from all patients. The study was conducted in accordance with the guidelines of the World Medical Association Declaration of Helsinki, and the Institutional ethical committee. The CONSORT guidelines (2010) for randomised trials were followed, and the PRIRATE checklist was respected (Data S1).

### 2.2 Sample Size Estimation

The current study was designed as a binary outcome superiority trial. The sample size calculation was based on the expected negative bacterial cultures after dressing the root canals with the test versus the control slurry (after the interim dressing). According to a previous study done under similar conditions, a proportion of negative growth of 0.2 (6 out of 30) was expected after the cleaning and shaping procedure (Ballal et al. 2019). The pure Ca(OH)_2_ suspension was not expected to alter that proportion (Waltimo et al. 2005; Carvalho et al. 2020), whilst the counterpart containing NaOCl was thought to result in a respective negative growth proportion of 0.5. Based on these proportions, an a priori sample size calculation for two independent groups (Fisher's exact test) was performed https://www.sealedenvelope.com/power/binary‐superiority/(G*Power 3.1, Heinrich Heine Universität Düsseldorf, Germany) with an alpha‐type error of 5% and a power of 80%, resulting in a total sample size of 88 (44 per group). Considering potential missing values due to procedural complications or non‐compliance of patients, the total sample size was set to 110 (*n* = 55).

### 2.3 Inclusion and Exclusion Criteria

The inclusion criteria were medically healthy and non‐pregnant patients aged above 18 years attending the Department of Conservative Dentistry and Endodontics, presenting a single‐rooted tooth with a pulpal diagnosis of necrosis and an apical diagnosis of asymptomatic apical periodontitis (American Association of Endodontists 2009). The diagnosis was established according to the patient's history, clinical inspection, palpation, tenderness to percussion, pulpal sensibility testing, probing depth and radiographic examination. Patients were not eligible for the study if they were not willing or able to give informed consent, or if they presented with: (i) a chronic condition requiring the intake of anti‐inflammatory/antibiotic drugs, (ii) a tooth which could not be isolated by rubber dam properly, (iii) a non‐restorable tooth. The patients included were selected from individuals seen at the outpatient clinic over the time course of this study. They all gave informed consent to participate.

### 2.4 Clinical Procedures

**FIGURE 1:**
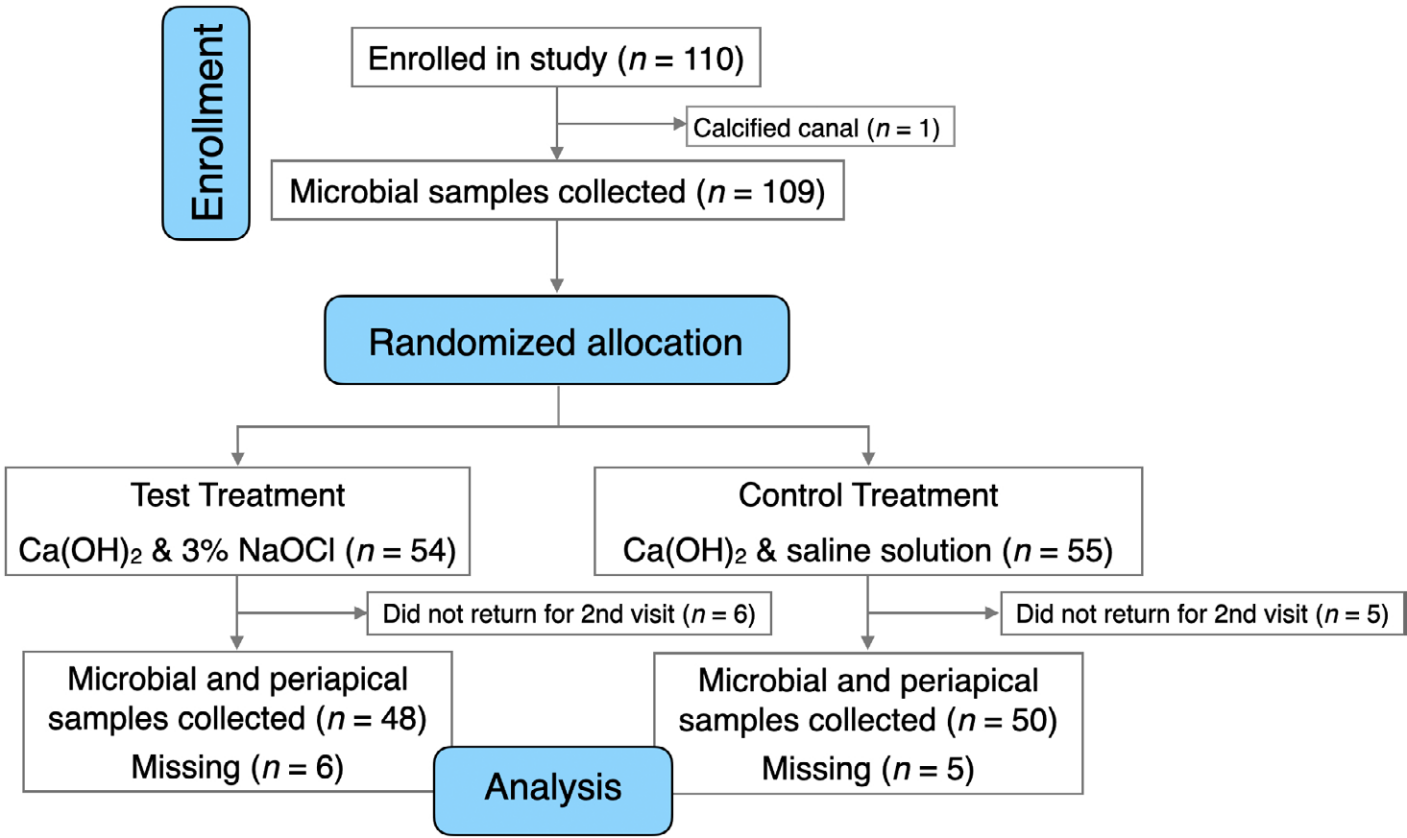
The Consolidated Standards of Reporting Trials (CONSORT) flow diagram depicting the journey of the eligible patients from enrollment to final assessment in the two treatment arms. There was one single‐rooted tooth in need of root canal treatment with each patient. [Correction added on 12 December 2025, after first online publication: the Figure 1 has been updated in this version.]

One hundred and ten patients meeting the inclusion criteria were selected. A CONSORT flow diagram outlining the treatment methodology is represented in Figure 1. Patients were randomly divided into two arms based on the type of intracanal medicament used. Random sequence generation was performed using a computer‐generated randomization process (www.randomizer.org). Allocation concealment was achieved using a sequentially numbered opaque sealed envelope (SNOSE) approach with a 1:1 allocation ratio. One researcher (JV) who was not part of this study, picked a closed envelope containing the instruction to use Ca(OH)_2_ powder (Pulpdent, Watertown, MA, USA), either mixed with physiological saline solution (Fresenius Kabi, Bad Homburg, Germany) or with 3% NaOCl (Vista Apex, Racine, WI, USA). Patients and the laboratory personnel performing the microbial analysis were blinded to the test medicaments, but not the operator who mixed the preparations chair‐side.

Teeth in both the groups were treated according to a standard protocol. They were isolated with a rubber dam (Coltène, Altstätten, Switzerland). The operating field was disinfected by swabbing with 30% hydrogen peroxide, followed by 5% tincture of iodine (Möller 1966). Subsequently, the access cavity was prepared using a sterile diamond‐coated bur (Horico Dental, Berlin, Germany). Working length was determined using an electronic apex locator (Root ZX; J. Morita, Kyoto, Japan). Patency of the root canal was achieved using an ISO‐size 10 K‐file (Dentsply Sirona, Ballaigues, Switzerland). The canal was then enlarged apically to size 20 using hand instrumentation. At this point, the pre‐treatment microbial sample was collected by placing a sterile ISO‐size 20 paper point (Dentsply Sirona) to working length for 1 min. The paper point was immediately placed inside a sterile Eppendorf tube (Merck, Darmstadt, Germany) containing 1 mL of reduced transport fluid (Syed and Loesche 1972). This procedure was repeated with a second paper point, which was added to the same tube. The tubes for microbial growth assessment were transferred to the lab immediately and processed the same day (see below).

Subsequently, the root canal was apically enlarged to 0.3 mm (F3) using ProTaper Universal instruments (Dentsply Sirona). Between each instrument change, the root canal was irrigated with 5 mL of 3% NaOCl during 1 min. Hence, a total of 25 mL of the irrigating solution was used. Irrigation was performed using a 30‐gauge side‐vented needle (Vista Apex), which was kept 1 mm short of the working length. Once the shaping procedure was completed, the root canal was flushed with 5 mL of 0.5% sodium thiosulfate (Merck) for 1 min, followed by 5 mL of distilled water for 1 min, to avoid potential carry‐over effects by NaOCl remnants. Then a second microbial sample was obtained as described.

Once the chemo‐mechanical preparation was completed, the pre‐randomised card was taken from the envelope to indicate the type of intra‐canal medicament to be used. In the test group, canals were filled with a slurry of Ca(OH)_2_ powder mixed with 3% NaOCl (Vista Apex). In the control group, the canal was filled with a similar Ca(OH)_2_ slurry, obtained by mixing the powder with physiological saline solution. Both these slurries were mixed at a solid‐to‐liquid ratio of 1:1.5 (wt/vol) to form a thin suspension for maximum efficacy (Behnen et al. 2001), so that they could just be administered using a size‐25 spiral filler (Lentulo, Dentsply Sirona). The access cavity of the tooth was then temporised using a calcium sulphate material (Cavit W, 3 M ESPE, Seefeld, Germany).

Patients were recalled after 1 week. On the recall visit, the tooth was again isolated with a rubber dam and the operating field was disinfected as described above. The previously sampled root canal was re‐entered and irrigated with 20 mL of 10% citric acid for 5 min to stop the Ca(OH)_2_ (Möller 1966) as well as the NaOCl effects by reducing possibly present hypochlorite remnants (Baumgartner and Ibay 1987). Then the canal was flushed with 5 mL of sterile physiological saline solution for 1 min. Subsequently a third (post‐dressing) microbial sample was collected as described. A periapical fluid sample was collected by introducing a fine sterile size‐20 paper point 2 mm beyond the apex for 1 min (Shimauchi et al. 1996). This procedure was repeated once with a second paper point. The paper points were placed in a sterile Eppendorf tube containing 2 mL of sterile physiological saline solution and immediately transferred to a −80°C freezer until further processing.

After the final sampling procedure, root canals were irrigated with 5 mL of 3% NaOCl (Vista Apex) followed by 5 mL of 17% EDTA (Vista Apex) for 1 min. Then they were filled with AH Plus sealer (Dentsply Sirona) and matched gutta‐percha points (Dentsply Sirona), and a post‐endodontic restoration was performed.

### 2.5 Microbial Analysis

In the clinic, samples were inoculated in reduced transport fluid (Syed and Loesche 1972) and immediately transported to the microbiology laboratory for processing. Upon receipt, tubes were vortexed, and quantitative cultures were performed from a 10‐fold dilution series. Aliquots were plated on pre‐reduced 5% sheep blood agar (BD, Becton Dickinson, Heidelberg, Germany). The plates were then incubated at 37°C for 72 h in an anaerobic environment using a Whitley A35 Anaerobic Workstation (Don Whitley Scientific, Shipley, UK). Following incubation, the bacterial colonies were counted, and colony‐forming units (CFUs/ml of transport fluid = CFUs/root canal) were calculated for each specimen. The detection limit was 100 CFUs/ml, as 10 μL of transport fluid was plated before dilution.

### 2.6 Assessment of Periapical Fluid Samples

Paper points were collected in individual sterile micro‐centrifugation tubes and stored at −80°C until analysis. On the day of analysis, the samples were eluted in 2 mL of sterile phosphate buffered saline (pH 7.2) by centrifuging at 2000 × g for 30 min at 4°C. The supernatant was collected and used for analysis. The levels of MMP‐9 were measured using a commercially available specific enzyme‐linked immunosorbent assay (ELISA) kit (Krishgen Biosystems, Mumbai, India). MMP levels were normalized to total protein (TP) in each sample. TP was determined using the Biuret method (Burtis and Ashwood 1999) against a standard series of bovine serum albumin.

### 2.7 Data Presentation and Analysis

Data were analysed using a statistics program (JMP Pro 17, Cary, NC, USA). Binary categorical data were compared between groups using Fisher's exact test (two‐tailed). Categorical data with more than two categories were compared using Pearson's chi‐squared test. Continuous data relating to anaerobic CFU counts and MMP‐9/TP levels were skewed (Shapiro–Wilk test) and compared using non‐parametric methods (Mann–Whitney *U* test for independent samples and Wilcoxon signed‐rank test for dependent counterparts). These values are presented as medians and inter‐quartile ranges (IQRs). Patient age was normally distributed and compared between test and control groups using Student's *t*‐test. These values are presented as means and standard deviations. The alpha‐type error was set at 5% (*p* < 0.05).

## 3 Results

The randomization process of this study resulted in statistically similar patient and tooth type distribution between the test and the control group (Table 1).

**TABLE 1:** Comparison of the 109 teeth receiving random allocation to an inter‐visit dressing with a calcium hydroxide slurry prepared with the respective solution.

	2.5% NaOCl (*n* = 54)	Physiological saline solution (*n* = 55)	*p* value
Patient gender	22 female/32 male	29 female/26 male	0.251^a^
Patient age	42 ± 14 years	40 ± 13 years	0.552^b^
Jaw	26 mandible/28 maxilla	19 mandible/36 maxilla	0.176^a^
Tooth type	9 C/26 I/19 PM	5 C/24 I/26 PM	0.316^c^

Abbreviations: C, canine; I, incisor; PM, premolar.

^a^ Fisher's exact test, two‐tailed.

^b^ Student's *t*‐test, two‐tailed.

^c^ Pearson's chi‐squared test.

From the 110 patients enrolled, no microbiological or periapical fluid samples could be obtained from 1 individual because the canal was calcified. In 11 study participants, no post‐dressing samples could be obtained because the patients did not return for the second visit (Figure 1).

**FIGURE 2:**
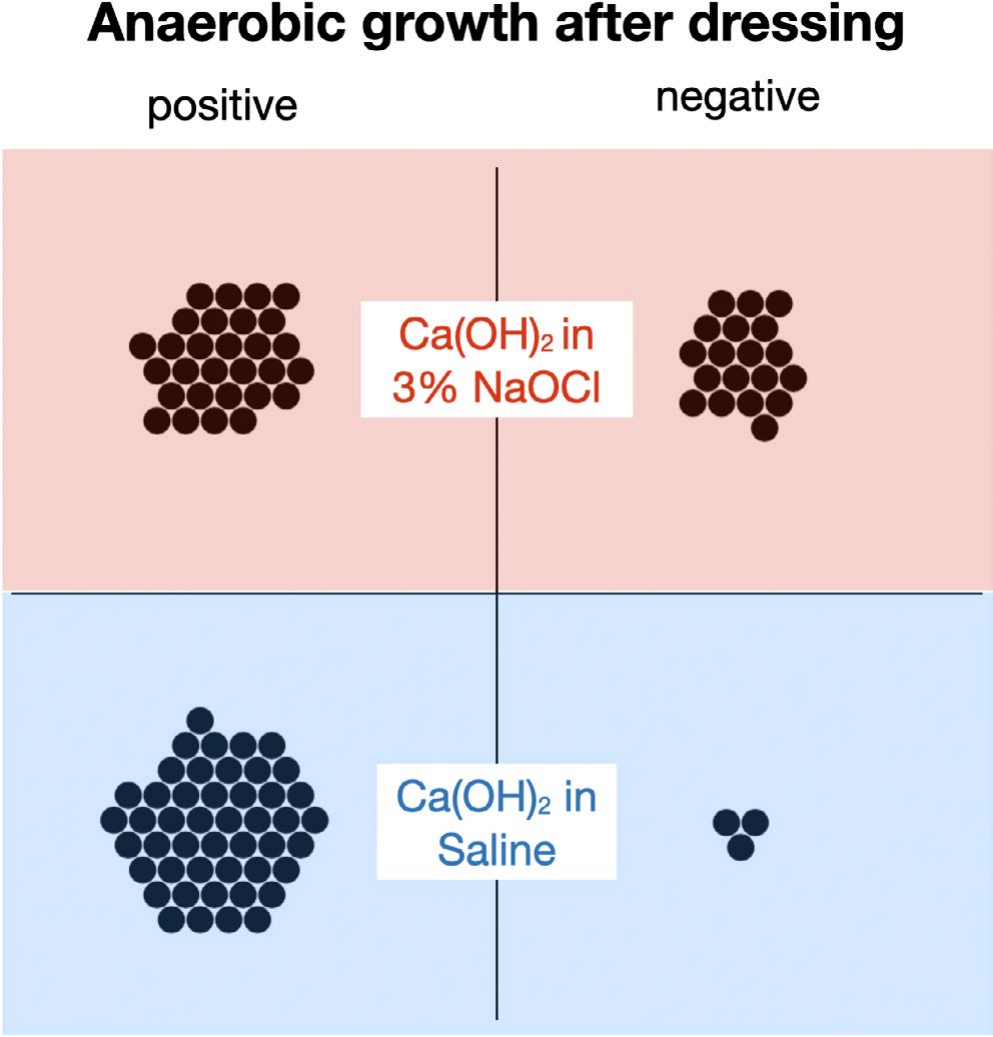
Dot plot depicting individual samples (dots) testing positive and negative for anaerobic culture after root canals had been dressed with a slurry made from pure Ca(OH)_2_ powder with 3% NaOCl (test group, red) or Ca(OH)_2_ powder with physiological saline solution (control group, blue) for 1 week. [Correction added on 12 December 2025, after first online publication: the Figure 2 has been updated in this version.]

There were no significant differences between treatment groups regarding CFU counts before administering the test or control Ca(OH)_2_ slurry (Table 2, *p* > 0.05). Instrumentation and irrigation resulted in a highly significant reduction of CFU counts (*p* < 0.001), again with no difference between groups (Table 2). However, all the samples remained positive for anaerobic growth. Post‐dressing samples tested negative for anaerobic growth in 40% (19 of 48) of the root canals dressed with a Ca(OH)_2_/NaOCl slurry, compared to 6% (3 of 50 total samples) dressed with Ca(OH)_2_/saline (Figure 2 *, p* < 0.001). Moreover, the inter‐visit Ca(OH)_2_/NaOCl dressing resulted in significantly (*p* < 0.001) fewer CFU counts compared to the Ca(OH)_2_/saline counterpart (Table 2).

**TABLE 2:** Comparison of the culturable bacteria (CFU/mL of transport medium, median values and inter‐quartile ranges) recovered from root canal systems after different treatment steps according to the solution the Ca(OH)_2_ slurry was prepared with (administered between S2 and S3).

Treatment step	2.5% NaOCl	PSS	*p* value^a^
Canal negotiation	2 × 10^4^ (1 × 10^4^–6.75 × 10^4^)	3 × 10^4^ (1 × 10^4^–5 × 10^4^)	0.483
Before dressing	9.5 × 10^2^ (2 × 10^2^–2.25 × 10^3^)	3 × 10^2^ (2 × 10^2^–5 × 10^3^)	0.686
After dressing	1 × 10^2^ (NG—1 × 10^2^)	1 × 10^2^(1 × 10^2^–5 × 10^2^)	< 0.001

Abbreviation: PSS, physiological saline solution (0.9% NaCl).

^a^ Wilcoxon signed rank test; NG = no growth.

MMP‐9/TP levels in the periapical fluid were similar between the two experimental groups after the root dressing period, 2.5 (IQR = 4.5) ng/g in the Ca(OH)_2_/NaOCl versus 1.4 (IQR = 4.6) ng/g in the Ca(OH)_2_/saline group (*p* > 0.05).

## 4 Discussion

The current study assessed the compatibility between Ca(OH)_2_ and NaOCl in a clinical setting. Even though mixing Ca(OH)_2_ powder with the (naturally sterile) NaOCl solution used for root canal irrigation as done here is taught in some dental schools, there have been, hitherto, no clinical data supporting this concept. The results of the present study demonstrated that combined Ca(OH)_2_/NaOCl dressing significantly reduced the microbial load, compared to a conventional slurry prepared with saline solution, in the root canals of teeth with primary apical periodontitis. Hence, the null hypothesis of this study was rejected.

This study is limited by multiple factors. Whilst the primary outcome under investigation has been linked to clinical success (Sjögren et al. 1997), it is still just a surrogate. Whether the relatively minor yet highly significant differences in viable counts between the two calcium hydroxide slurries under investigation would also result in different clinical outcomes is therefore questionable (Peters and Wesselink 2002). Root canal treatment is a multi‐step procedure, and differences in root canal disinfection can be compensated by entombing the surviving micro‐organisms in a tight root filling. A further limitation of this study is the fact that not all the treatment steps performed here are commonly done in clinics. A stop solution was used, and canals were also irrigated with citric acid before the final sampling, which could have reduced viable counts (Smith and Wayman 1986). Furthermore, no control sample was taken before accessing the canal system (Möller 1966). Cavit, which was used as a temporary filling for the interim, is not necessarily bacteria‐tight (Beach et al. 1996), although care was exercised to place it in adequate thickness (Kampfer et al. 2007). However, this being a randomised trial with a relatively high number of observations, factors potentially inducing bias can be expected to be similarly distributed between groups, and the result can therefore still be considered to be valid.

This study used a traditional culture‐based approach to assess the disinfecting power of the Ca(OH)_2_ slurries under investigation. The focus was on anaerobic growth. While the evaluation of anaerobic bacterial survival is a solid measure of endodontic treatment, molecular studies have shown that it is the facultative Gram‐positive bacteria, especially from the phylum Firmicutes, that are persistent in root canals following chemo‐mechanical preparation (Siqueira Jr. et al. 2024). This is a further limitation of the current research, and a call to re‐investigate the issue under investigation using contemporary molecular methods.

To monitor inflammatory changes in periapical tissues, periapical tissue fluid samples were taken and assessed for MMP‐9, a neutrophil marker (Zehnder and Belibasakis 2022). This marker has been shown to react to a reduction in the intracanal bioburden in previous studies (Ballal et al. 2019), and was used here to detect potential untoward effects of the NaOCl. As could be expected by the short‐lived presence of the active chlorine in the NaOCl‐containing dressing, no such non‐desirable effects were observed. On the other hand, there was also no positive/desired effect of the presence of NaOCl in root canal dressing regarding this outcome. While measuring MMP‐9 would be a good measure of tissue breakdown, it may not tell the full story. Future studies on periapical biomarkers may have to assess more entities in periapical fluid to identify molecules with a higher diagnostic value (Matos‐Sousa et al. 2024).

This study revolved around a clinically relevant question, and that is the time a clinician has to spend with the patient to obtain optimal results. As instrumenting systems have become more and more efficient, their antimicrobial effect has not (Dalton et al. 1998). The average time for instrumentation of a mesial canal in a mandibular molar can be a mere 38 s when a single file in reciprocating motion is used (Paqué et al. 2011). This poses an ethical dilemma to the practitioner. Should he or she fill the root canal system immediately after instrumentation, irrigate copiously and activate the irrigating solution, or place an inter‐visit dressing? To fill immediately is most likely not a good idea, yet it is probably done more often than we wish to think, as it is economically interesting for the dentist/endodontist rendering the service and cannot unequivocally be monitored on the post‐obturation periapical radiograph. A considerate clinician, however, can follow different paths to make extra efforts towards debridement and disinfection of the root canal system after instrumentation has been completed. The focus in research has been on irrigation and activation of the NaOCl solution (Caputa et al. 2019). To let the NaOCl exert its effects passively in the root canal system (Ragnarsson et al. 2015) has not gained the attention it probably deserves. The only solution that has been assessed for its antimicrobial effect if placed in the root canal passively has been iodine potassium iodide (Kvist et al. 2004). A recent study on experimentally infected human molars found that whilst the choice of instrumenting system does not necessarily influence root canal disinfection, the time the NaOCl is allowed to act in the root canal system clearly does (Gazzaneo et al. 2019). This is in line with studies on anaerobic oral biofilm: NaOCl is the most efficient antibiofilm agent among common endodontic irrigants, yet its effect is time‐dependent (Stojicic et al. 2013).

The current results are in line with previously published data, both using culture (Waltimo et al. 2005) and culture‐independent techniques (Carvalho et al. 2020). These results all reveal that the more time is spent irrigating and disinfecting the root canal system, the higher the microbial reduction. What was new in this study was that the chemical compatibility of Ca(OH)_2_ and NaOCl was taken into consideration. Whether an intra‐visit application of plain NaOCl would lead to a similar reduction in anaerobic counts was not investigated and could be studied in future experiments.

## 5 Conclusions

Under the limitations of the current study, there was an additive antimicrobial effect between Ca(OH)_2_ and NaOCl when administered as an inter‐visit dressing. MMP‐9/TP levels in the periapical fluid were similar in root canals dressed with Ca(OH)_2_ slurries mixed with NaOCl or saline. This observation could be useful when timing/planning treatment sessions in endodontic practice.

## Author Contributions

N.V.B. and M.Z. were involved in conception and design, data analysis, and drafting the manuscript. N.R., P.A.S., V.A., and V.R.B. were involved in data acquisition. All authors critically revised the manuscript and gave their final approval.

## Conflicts of Interest

The authors declare no conflicts of interest.

## Supporting information


**Data S1:** Supplementary Information.

## Data Availability

The data that support the findings of this study are available from the corresponding author upon reasonable request.
